# Comparison of Facebook, Google Ads, and Reddit for the Recruitment of People Who Considered but Did Not Obtain Abortion Care in the United States: Cross-sectional Survey

**DOI:** 10.2196/22854

**Published:** 2021-02-24

**Authors:** Heidi Moseson, Alexandra Wollum, Jane W Seymour, Carmela Zuniga, Terri-Ann Thompson, Caitlin Gerdts

**Affiliations:** 1 Ibis Reproductive Health Oakland, CA United States; 2 Ibis Reproductive Health Cambridge, MA United States

**Keywords:** abortion, induced, abortion seekers, abortion surveys, bias, selection, pregnancy, unplanned, research subject recruitment, reproductive health, social media, social stigma

## Abstract

**Background:**

In the United States, abortion access is restricted by numerous logistical, financial, social, and policy barriers. Most studies on abortion-seeking experiences in the United States have recruited participants from abortion clinics. However, clinic-based recruitment strategies fail to capture the experiences of people who consider an abortion but do not make it to an abortion clinic. Research indicates that many people search for abortion information on the web; however, web-based recruitment remains underutilized in abortion research.

**Objective:**

This study aims to establish the feasibility of using Facebook, Google Ads, and Reddit as recruitment platforms for a study on abortion-seeking experiences in the United States.

**Methods:**

From August to September 2018, we posted recruitment advertisements for a survey about abortion-seeking experiences through Facebook, Google Ads, and Reddit. Eligible participants were US residents aged 15-49 years who had been pregnant in the past 5 years and had considered abortion for a pregnancy in this period but did not abort. For each platform, we recorded staff time to develop advertisements and manage recruitment, as well as costs related to advertisement buys and social marketing firm support. We summarized the number of views and clicks for each advertisement where possible, and we calculated metrics related to cost per recruited participant and recruitment rate by week for each platform. We assessed differences across platforms using the chi-square and Kruskal-Wallis tests.

**Results:**

Overall, study advertisements received 77,464 views in the 1-month period (from Facebook and Google; information not available for Reddit) and 2808 study page views. After clicking on the advertisements, there were 1254 initiations of the eligibility screening survey, which resulted in 98 eligible survey participants (75 recruited from Facebook, 14 from Google Ads, and 9 from Reddit). The cost for each eligible participant in each platform was US $49.48 for Facebook, US $265.93 for Google Ads, and US $182.78 for Reddit. A total of 84% (66/79) of those who screened eligible from Facebook completed the short survey compared with 73% (8/11) of those who screened eligible from Reddit and 13% (7/53) of those who screened eligible from Google Ads.

**Conclusions:**

These results suggest that Facebook advertisements may be the most time- and cost-effective strategy to recruit people who considered but did not obtain an abortion in the United States. Adapting and implementing Facebook-based recruitment strategies for research on abortion access could facilitate a more complete understanding of the barriers to abortion care in the United States.

## Introduction

### Background

Abortion is a safe and effective essential reproductive health service with social, economic, and physical benefits for those who wish to access care and are able to do so [1-7]. An estimated 862,320 abortions were provided in clinical settings in the United States in 2017, a decline of 7% since 2014 [8]. Due to well-established legal, financial, logistical, social, and other barriers [9-11], many people in the United States are not able to access abortion care [12]. The studies that have identified these barriers to abortion services have recruited participants almost exclusively from abortion clinics. However, this clinic-based sampling mechanism fails to account for pregnant people who want an abortion but are unable to make it to an abortion clinic [13] and may consequently underestimate the barriers and burdens that people face in obtaining abortion care.

As a strategy to address this recruitment gap, we reviewed the published literature on recruitment methods for difficult-to-target populations and evaluated their potential adaptability to the abortion research context. On the basis of this review, we hypothesized that web-based recruitment methods might enable us to reach people who have not traditionally been included in studies of abortion access. The geographic reach of the internet and social media, their ability to target specific population groups, and the privacy conferred by web-based data collection make these methods compelling options for abortion research in the United States. In January 2018, the Pew Research Center found that 88% of all women aged between 18 and 29 years and 78% of women aged between 30 and 49 years used at least one type of social media [14], and sociological research has found high social media engagement among transgender and nonbinary people assigned female at birth [15,16]. Given the widespread use of social media among these groups, these platforms may provide a reasonably complete sampling frame for the population of interest—pregnant people who considered but did not obtain an abortion.

### Objectives

Prior studies have successfully recruited people of reproductive age using web-based methods [17-19], including 1 study that recruited 1235 people in 1 month using a Google Ads campaign related to self-managed abortion [20] and another study that similarly used Google Ads to recruit 1706 pregnant people searching for information on abortion over 9 months [21]. In this study, we aim to test the feasibility of recruiting participants through 3 different web platforms and to compare each platform’s performance in recruiting a sample of people in the United States who considered but did not obtain an abortion. We sought to answer the following research question: Are Facebook, Google Ads, and Reddit able to recruit people who considered but did not obtain an abortion? Specifically, for each platform, we wanted to measure the cost of recruiting eligible research participants and how many eligible recruits could be enrolled per week, and to compare these values across the three platforms. On the basis of the limited research available at the time of the study design, we hypothesized that Google Ads would be the most successful [20]. Beyond comparison across the individual platforms, we also wanted to explore (1) how advertisement image characteristics related to viewer engagement with the study and (2) social network questions to gauge the feasibility of a future respondent-driven sampling study among this target population.

## Methods

### Recruitment

This study was approved by the Allendale Investigational Review Board in the United States. After conducting a systematic review of the peer-reviewed literature on nontraditional recruitment methods (results forthcoming), we selected 3 methods with the potential to recruit the target population: Facebook (including Instagram), Google Ads, and Reddit (one thread on birth control and another on menstruation). Over a 1-month period, between August 15, 2018, and September 15, 2018, we posted advertisements in English and Spanish on these platforms for a survey of experiences with unplanned pregnancy. All advertisements mentioned that participants would be entered into a raffle for the chance of winning a US $50 gift card. Modeling a recent study on self-managed abortion [22], we chose a 1-month pilot period recruitment window, which, though brief, would allow the detection of some variation in recruitment by week. A recruitment firm, BUMP Digital Marketing [23], managed the posting and purchasing of advertisements through Facebook and Google Ads, and study authors managed the Reddit campaigns. A background Facebook algorithm determined which advertisements were displayed on Facebook and Instagram based on user engagement with the early displays of each advertisement. For the remainder of this paper, we report both Facebook and Instagram results as *Facebook*.

Recruitment proceeded across all 3 platforms in the following steps: individuals who clicked on a study advertisement were directed to a study-specific web page with additional information about the study and a link to the eligibility screening questionnaire and informed consent materials. The screening questionnaire identified eligible participants: those aged 15-49 years, English- or Spanish-speaking residents of the United States, and those who had been pregnant in the past 5 years but had not visited an abortion clinic or obtained a wanted abortion in that period. In addition, eligible participants were those who responded *yes* to both of the following questions: “Did you consider abortion for any of these pregnancies, even for just one second?” and “If it had been available to you, could abortion have potentially been the best option for any of these pregnancies?” Those who consented were directed to a short web survey that collected data on pregnancy and abortion-seeking experience.

### Data Collection

We tracked staff time and overall costs, recruitment strategy performance metrics, and the number of eligible recruits by platform. To estimate the total costs for each recruitment approach, the study team recorded the amount of staff time spent designing the advertisements for the launch of the study, the cost of the advertisements themselves, and the cost of the marketing firm’s services. To measure recruitment strategy performance, the study team collected data on (1) impressions, defined as the number of times an advertisement was displayed on the platform; (2) reach, defined as the number of unique users to whom advertisements were displayed on each platform (when available); (3) the number of people who viewed the study website (website view *conversions*); (4) the number of people who began and completed the screening questions; (5) the number of people who were eligible; (6) the number of people who consented to participate; and (7) the number of people who began and completed the survey, overall and by platform. In addition, the study team tracked the specific advertisements that brought people to the study page, the language in which participants viewed the study material, and each participant’s internet protocol (IP) address (for identification of potential duplicate entries).

The survey was programmed and fielded in Qualtrics and included open- and closed-ended questions. Survey questions assessed experiences with unwanted pregnancy; interest in abortion; barriers to abortion care; knowledge of others who had not obtained wanted abortion care; and sociodemographic characteristics, including state of residence, number of children, annual household income, work status, gender, education, race and ethnicity, and health insurance coverage. Participants who completed the survey were entered into a raffle for a US $50 gift card.

### Statistical Analysis

The total staff time spent on advertisement development was multiplied by an hourly wage of US $30 per hour to estimate costs per platform. To estimate the total cost per platform, we summed all the costs. For some of the tasks (eg, advertisement text development, advertisement text translation, and landing page development), the hours worked or dollars charged were counted for each of the platforms individually, as the task would have been necessary for each platform had we piloted only 1 method, although the amount was only spent once. For other tasks (eg, translation of keywords and image finding), staff time, marketing expenses, and advertisement buys were attributed only to the relevant platform. Marketing firm fees included study website development in 2 languages, building an advertising campaign, and advertising campaign maintenance costs. To estimate the cost per eligible survey, we divided the total cost by platform by the number of eligible surveys completed from that platform.

To assess the performance of each of the 3 recruitment platforms, descriptive analyses of the number of views and clicks for each advertisement and the number of screeners and survey initiations and completions were calculated automatically by analytics on each platform (Facebook and Google) or manually via submissions in Qualtrics (Reddit). The research team manually identified and removed multiple survey submissions from the same IP address but could not do so for clicks or website views. The first survey submission by a unique IP address was chosen by default as the submission to include in the analysis, unless the repeat submission was clearly made only to correct an error in response to the screener questions (eg, entering age *9* in the first submission and *19* in the second submission). To assess the recruitment rate, we calculated the total number of eligible participants recruited by week and the mean and SD of recruits per week from each platform.

To understand whether the *tone* of the image used in an advertisement was associated with engagement, we compared engagement statistics (impression, reach, study website views, and conversions) between 2 advertisements with the same text but different images—one presenting a person looking directly at the camera with confidence and the other showing the profile of someone looking downward out of a window with a more somber expression. We present comparisons of the performance of these 2 advertisements by the language of the advertisement (English and Spanish) among low-income Facebook users; we targeted advertisements toward low-income Facebook users as they might be most likely to face barriers to abortion care.

Additional analyses assessed the distribution of participants’ sociodemographic characteristics (age and language) overall and by study recruitment platform, as well as concordance between participant self-report of the web-based platform that led them to the study web page and electronic data on the recruitment platform actually used, with chi-square and Kruskal-Wallis tests for categorical and continuous variables, respectively. The mean number of people known by each respondent who failed to obtain a desired abortion for a previous pregnancy and the mean number to whom the respondent would feel comfortable giving information about the study were calculated to explore the feasibility of using web-based methods to recruit participants for future research using a social network-based approach. Standard deviations (SDs) and ranges were also calculated for both these estimates. All analyses were conducted using Stata version 15.

## Results

### Advertisement Views and Engagement

**Figure 1:**
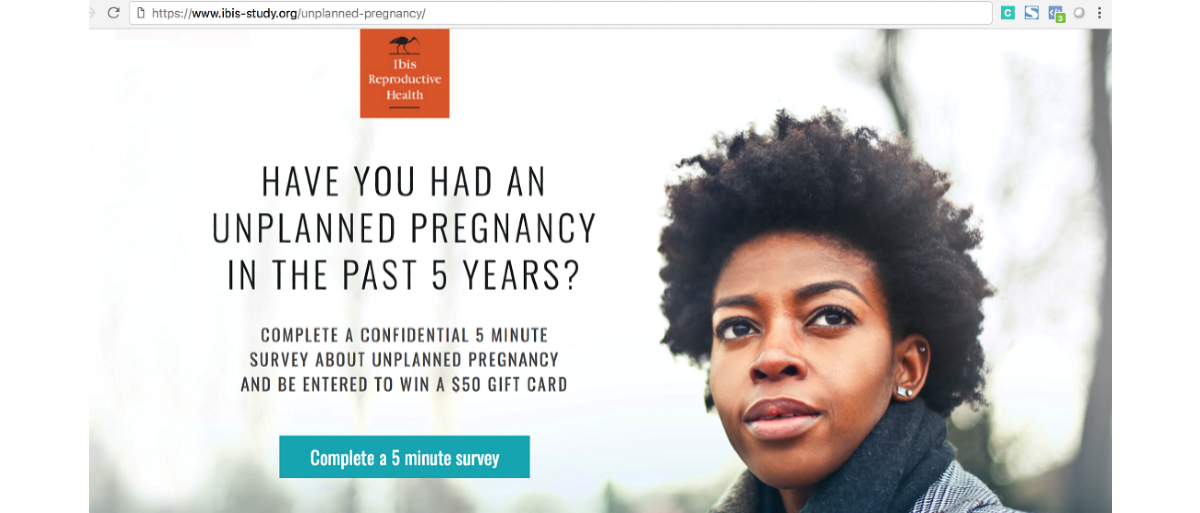
Study website (landing page) displaying study information and offering links to click-through to the survey.

**Figure 2:**
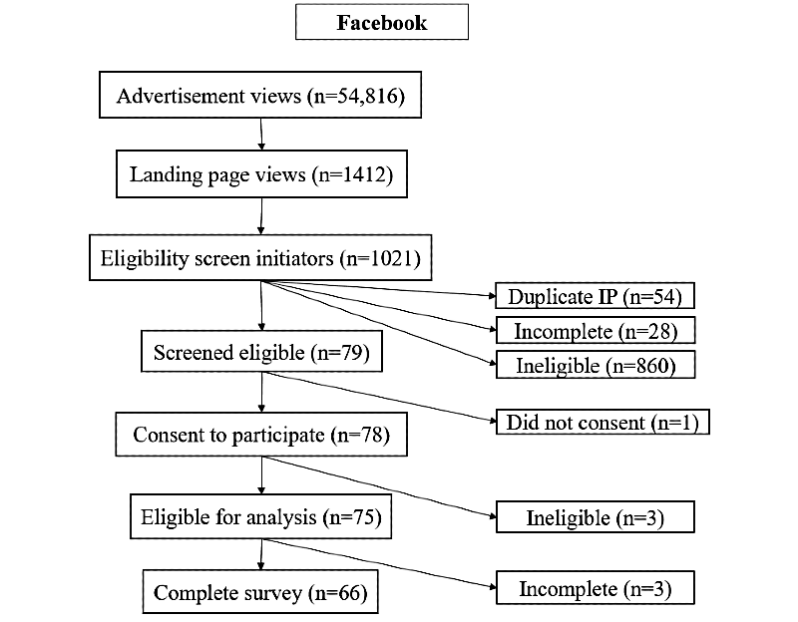
Summary flowchart displaying engagement and response pathways for all study advertisements posted on Facebook. IP: internet protocol.

**Figure 3:**
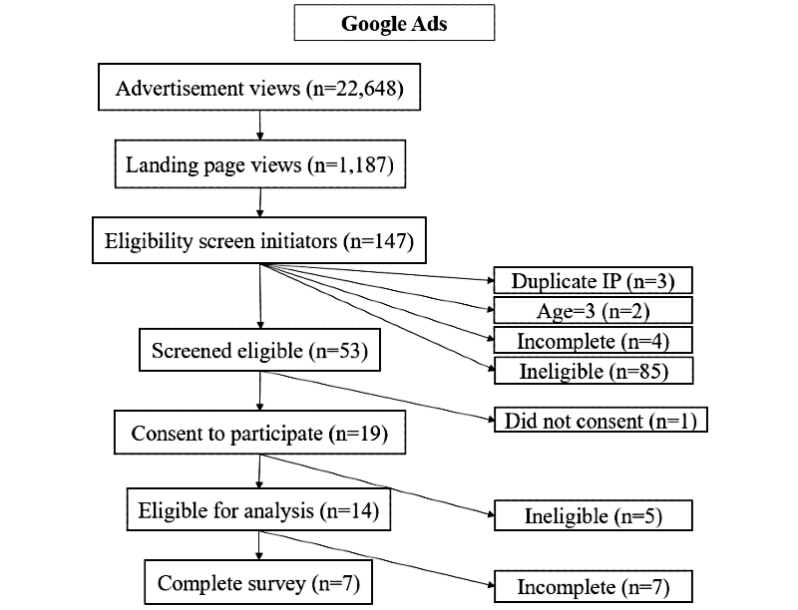
Summary flowchart displaying engagement and response pathways for all study advertisements posted on Google Ads. IP: internet protocol.

**Figure 4:**
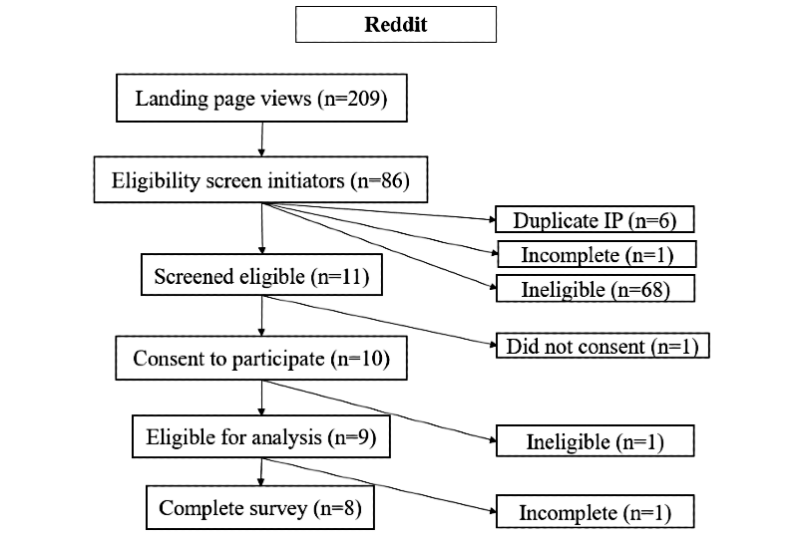
Summary flowchart displaying engagement and response pathways for all study advertisements posted on Reddit. IP: internet protocol.

During the 1-month period, study advertisements received 77,464 known views, and the study website (or *landing page*; Figure 1) received 2808 views. Advertisement and study website views by platform are displayed in Figures 2-4. Google Ads had the highest conversion of advertisement views to study website views (1187/22,648, 5.24% overall), followed by Facebook (1412/54,816, 2.58% overall). These data were not available for Reddit.

**Figure 5:**
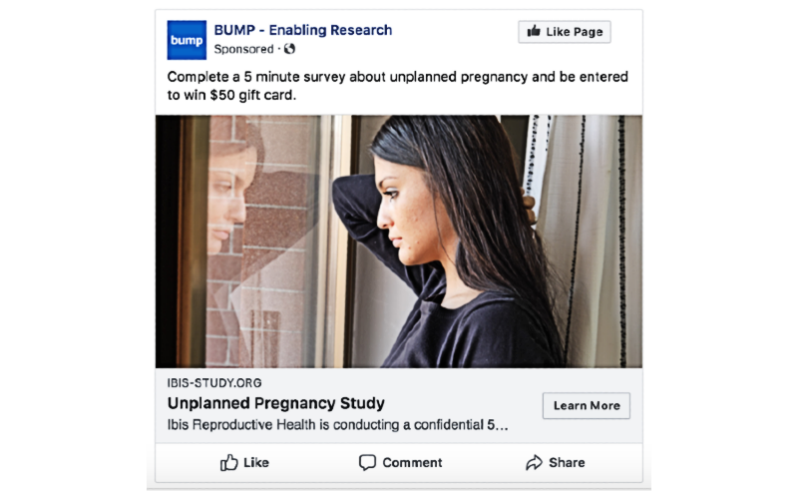
Screenshot of the study advertisement that received the most engagement of all English-language advertisements posted on Facebook.

**Figure 6:**
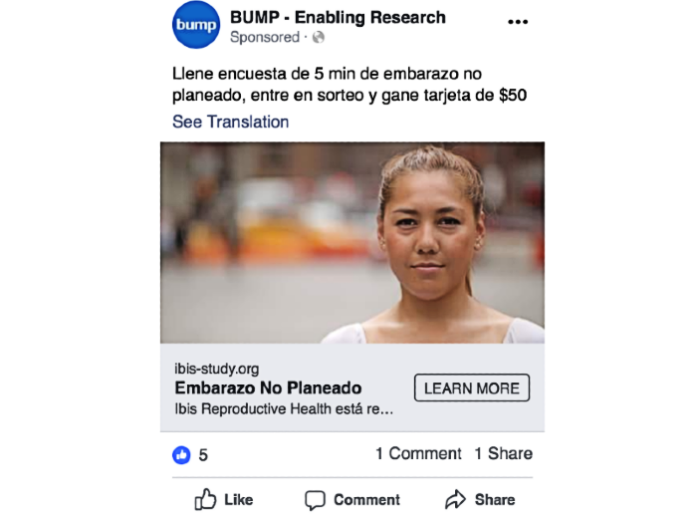
Screenshot of the study advertisement that received the most engagement of all Spanish-language advertisements posted on Facebook.

To understand individual advertisement reach, Facebook provided the most complete data. Table 1 displays information on the number of impressions, reach, and study website conversions for the top 2 advertisements in English (Figure 5) and Spanish (Figure 6). Among the English-language advertisements, the image in Figure 5 garnered the most engagement, whereas among the Spanish-language advertisements, the image in Figure 6 received the most engagement.

**Table 1:** Impressions, reach, and study website views for 2 study advertisements displayed on Facebook to 2 target audiences of low-income English speakers and low-income Spanish speakers. Models in both advertisements had dark hair and appeared to be aged between 20 and 40 years.

Target audience		Impressions^a^ (N)	Reach^b^ (N)	Unique study page views, n (%)	Conversions^c^, n (%)	Total cost (US $)	Cost per conversion (US $)
**Low-income English speakers**							
	Dark shirt, looking out of the window	22,768	13,973	398 (2.85)	310 (77.9)	216.76	0.70
	Light shirt, looking straight-on	16,665	10,157	303 (2.98)	248 (81.8)	155.66	0.63
**Low-income Spanish speakers**							
	Dark shirt, looking out of the window	4266	2572	23 (0.89)	11 (48)	39.08	3.55
	Light shirt, looking straight-on	16,614	7840	83 (1.06)	56 (68)	139.57	2.49

^a^Impressions refers to the number of times an advertisement was displayed on the platform.

^b^Reach refers to the number of unique users to whom the advertisement was displayed on the platform.

^c^Conversion refers to the number of people that clicked from the study website to the screener survey.

### Eligibility Screen Completions and Eligible Recruits by Platform

Study advertisement views led to 1254 completions of the eligibility screener from 1191 unique IP addresses (63 screeners were submitted from a duplicate IP address). Among those who viewed the study website, 68.48% (967/1412) of Facebook users completed the screening questionnaire, as compared with 11.96% (142/1187) of Google Ads recruits and 38.3% (80/209) of Reddit recruits.

After completing all screening questions, 11.4% (143/1254) of all unique screener initiators were eligible: 7.73% (79/1021) of Facebook recruits, 36.1% (53/147) of Google Ads users, and 13% (11/86) of Reddit users. Among screener respondents, 84.44% (1059/1254) reported that they were currently pregnant or had been pregnant in the last 5 years, and of these, 20.49% (217/1059) said that abortion would potentially have been the best option for one or more pregnancies if it had been accessible. The largest driver of ineligibility among screener initiators was a *no* response to whether the respondent had considered abortion for any of their recent pregnancies, even for just 1 second (605/1013, 59.72% of those who screened ineligible). The second largest driver of ineligibility was a *no* response to the question about whether abortion would have been the best option for any recent pregnancy if it had been available to them (206/1013, 20.34% of those who screened ineligible), followed by having visited a clinic or hospital to learn about options for the pregnancy (104/1013, 10.27% of those who screened ineligible). Age and recent pregnancy were not the major drivers of ineligibility.

In total, 68.5% (98/143) of eligible participants consented and completed at least some of the questions included in the main survey: 95% (75/79) of eligible Facebook respondents, 26% (14/53) of eligible Google Ads respondents, and 82% (9/11) of eligible Reddit respondents. Overall, 56.6% (81/143) of those who screened eligible went on to complete the entire survey (66 from Facebook, 7 from Google Ads, and 8 from Reddit). The recruitment rate was approximately 18 (SD 4) eligible subjects per week from Facebook, 3 (SD 1) per week from Google Ads, and 2 (SD 1) per week from Reddit.

Including staff time, marketing firm fees, and advertisement buys, the research team spent approximately US $5745 on recruitment efforts across all 3 platforms (Table 2). This translated to a total cost per platform of US $3711 for Facebook, US $3723 for Google Ads, and US $1645 for Reddit. The cost per eligible survey translated to US $49.48 for Facebook, US $265.93 for Google Ads, and US $182.78 for Reddit.

**Table 2:** Funds expended on recruitment efforts, including staff time, marketing firm fees, and advertisement buys, were recorded in 2018 US dollars.

Recruitment expenses	Recruitment platform		
	Facebook	Google Ads	Reddit
Staff time, min	240	210	290
Staff cost, US $	120	105	145
Advertisement buys, US $	791	818	0
Marketing firm fees, US $	2800	2800	1500
Total cost, US $	3711	3723	1645
Cost per eligible survey, US $	49.48	265.93	182.78

### Respondent Characteristics

Among the unique screener completions (n=1189), the average reported age was 29 years (SD 6.3; range 14-46 years). Age differed across the 3 recruitment platforms (Kruskal-Wallis *P*<.001); for Facebook, the average age was 30 years (SD 5.8; range 18-45 years); for Google Ads, the average age was 24 years (SD 7.1; range 14-46 years); and for Reddit, the average age was 23.8 years (SD 5; range 16-41 years). The language in which the screener was completed also differed across platforms (chi-square *P*<.001): 7.34% (75/1021) of the Facebook participants completed the screener in Spanish, 32.0% (47/147) of the Google Ads participants completed the screener in Spanish, and Reddit advertisements were posted in English only.

When asked about their social network, survey respondents reported knowing an average of 4 people who wanted an abortion but did not get one and were willing to share information about the study with an average of 2 of these people. Overall, 92.5% (99/107) of the survey participants accurately reported where they saw the study advertisement (Facebook, Google, or Reddit). All 100% (10/10) Reddit respondents accurately reported the referral platform, as did 99% (77/78) of Facebook respondents and 63% (12/19) of Google respondents.

## Discussion

### Principal Findings

We sought to advance the understanding of recruitment-based methods to address potential selection bias in research on abortion access. To do so, we piloted 3 web-based approaches to recruit a narrowly defined, difficult-to-target, and potentially stigmatized population that has not been included in traditional, clinic-based abortion research—people who self-identify as not having obtained an abortion for a recent pregnancy, despite having considered abortion and feeling that that abortion could have been the best option. We found that it is feasible and cost-effective to use web-based platforms—particularly Facebook, which balances cost and participant recruitment most efficiently—to recruit this specific population.

### Limitations

As with all research, this study has strengths and limitations. The recruitment methods tested were identified through a systematic review process to employ the methods most likely to succeed in our target population. To create the conditions for a fair test of each web-based platform’s ability to recruit our target population, we recruited through all 3 platforms simultaneously, using the same text for the advertisements where possible; conducted outreach in both English and Spanish; and tested multiple images in the advertisements. A professional social marketing firm assisted our study team, facilitating the creation and implementation of the most rigorous test possible of Facebook and Google Ads; however, nonmarketing professionals (the study team) managed the Reddit campaign. As a result, findings may differ if a marketing professional managed all recruitment streams.

The findings are limited in that we could not pilot the advertisement images before launching the full recruitment campaign. Due to a failure in the embedded tracking code, we could not assess which advertisements converted the most participants, as we could not differentiate participants by advertisement beyond the study website; this issue, however, has been remedied for future studies. We were also limited by a small budget for advertisements on Google Ads and Facebook and by our time frame, and we used only 2 subthreads within Reddit. To streamline the recruitment process, we collected little data on the population that visited the landing page and were screened, limiting our ability to comment on the differences between the individuals who were targeted by our advertisements but were not in our target population. An additional limitation is tied to the fact that we did not create a dedicated Instagram advertisement set or allocate money specifically to Instagram advertisements. Thus, because of low initial engagement, Facebook allocated nearly all of our advertisement dollars to Facebook and not Instagram; this resulted in very few advertisement impressions on Instagram, which limited our ability to assess recruitment performance via Instagram. Furthermore, although coverage of our target population among internet and social media users is estimated to be high, a better understanding of who does and does not use each platform will provide insight into how estimates from these web-based recruited samples should be interpreted. Given the timeline of research, another limitation is that there will always be a delay between when data are collected and study findings are shared—a delay during which the algorithms and systems used by these web-based platforms have continued to iterate, which could potentially shift findings in unknown ways. Finally, based on the decision to keep only the first survey submission from a given IP address, we may inadvertently have lost relevant study information provided in subsequent submissions.

### Comparison With Prior Work

Our results are consistent with a large proportion of prior studies that compared web-based recruitment strategies, including among hard-to-reach populations (although never within our specific target population), which found Facebook advertising to be the most effective method in terms of cost and absolute numbers. Exceptions included studies in which a specific platform was organized around a key characteristic of the target population, such as dating apps for gay and bisexual men (eg, Grindr) [24-26]. In a systematic review of 35 studies that used Facebook for recruitment, the average cost per participant was US $14.41 and the median number of participants recruited was 264 over a 3-month period (approximately 88 per month) [27], as compared with an average cost of US $49.48 for the 75 eligible, consented survey initiators recruited over 1 month in this study. Although some studies included in the review overrepresented the experiences of young, White, cisgender women, most resulted in study samples that mirrored national sociodemographic statistics [27], a finding that supports the potential use of Facebook-focused recruitment efforts for future studies.

Studies that used Google Ads, although fewer than those reporting on Facebook recruitment, have returned more variable results, with many recruiting higher numbers of participants than this study. A recent study on self-managed abortion recruited over 1200 participants in a 1-month period [22]. Another study that recruited pregnant people seeking information on abortion providers enrolled approximately 190 participants per month over 9 months [21], compared with the 143 who initiated our screener in that same period. A more recent study focused on those currently pregnant and searching for information on abortion providers reported an average cost (based on advertisement spend) of US $18.85 per completed baseline survey [21], as compared with US $58.43 (based on advertisement spend only, not including staff and social media firm time) for this study. It may be that (1) Google Ads is better suited to target currently pregnant people versus those who have been pregnant in the past 5 years, or (2) the difference could reflect the substantial variance in advertising budgets, or (3) the fact that participants in this study only had the chance of winning US $50, rather than guaranteed remuneration. It is worth noting that in this study, Google Ads recruited more Spanish speakers and younger people (as measured via click-throughs) than Facebook or Reddit. The few studies published using Reddit have returned results similar to our findings, including one that recruited 34 young men who have sex with men in a 2.5-month period [26]. We did not identify comparable cost comparison data from other Reddit studies.

### Recommendations

Given the consistency between the results presented here and in the prior literature, Facebook and Google Ads may be promising platforms for recruiting people who consider abortion in the United States but never make it to an abortion clinic. The click-through rates of our advertisements on Facebook and Google Ads were higher than the industry standard for both platforms [28], suggesting a high level of interest and engagement, although costs per eligible recruit were quite high with Google Ads. Our results also suggest that, to some extent, people share the experience of not having a wanted abortion within their social network, as indicated by the fact that 1 out of 5 participants knew someone in their network who had not obtained a wanted abortion in the past 5 years, and of these people, over half would be willing to share information about the study. This finding indicates an opportunity to pilot web-based referral methods to recruit this population.

Additional research, conducted on a larger scale over a longer time frame, is needed to build and expand upon the initial findings of this feasibility study. A larger study that recruits through Facebook and adds a dedicated Instagram-based recruitment arm, with structured incentives to encourage respondent-driven recruitment, could broaden the diversity of age, ethnicity, and level of education reached by the study advertisements. Relatedly, now that this study has established that these web-based platforms can successfully recruit from this narrowly defined population, future research should use these platforms with broader eligibility criteria to recruit people who considered abortion to explore experiences that differ across those who do and do not access abortion facilities. A larger budget (beyond the US $5745 spent for recruitment in this study) might dramatically increase the visibility and reach of the study advertisements beyond what was possible in this feasibility study. We recommend that future work carefully pilot test recruitment advertisement text and images and develop and embed tracking code so that participants can be followed from the initial advertisement view through survey completion and that researchers consider independently funded campaigns for each advertisement to test the performance of individual advertisements in a more controlled manner. Furthermore, researchers can consider the incorporation of complementary, free posts to Facebook groups and pages when relevant groups exist if the content of study messaging is not seen to be overtly stigmatizing or compromising participant privacy.

### Conclusions

Without data from people considering abortion (not just those who make it to an abortion clinic), we cannot know if and how our understanding of the scope and magnitude of barriers to abortion care is limited and what the true implications are for abortion-related programs, policies, and clinical practices. Results suggest that as we strive to better understand the full abortion-seeking experience, recruitment via Facebook may be a promising approach to sample from a more complete group of people who consider abortion. Our findings show the potential of web-based recruitment methods to address potential selection bias in research. We encourage researchers who study abortion access and those who work on other sensitive and/or stigmatized areas of population health to further explore and test the use of these methods and to consider how recruitment methods and sample selection may influence the conclusions of our research.

## Abbreviations

IP: internet protocol
